# High‐grade traumatic torso visceral injury with hemodynamic instability: effectiveness of transarterial embolization using n‐butyl cyanoacrylate

**DOI:** 10.1002/ams2.264

**Published:** 2017-03-06

**Authors:** Junya Tsurukiri, Shoichi Ohta, Akira Hoshiai, Hidefumi Sano, Eitaro Okumura, Nobuhiko Tsubouchi, Hiroyuki Konishi, Tetsuo Yukioka

**Affiliations:** ^1^ Emergency and Critical Care Medicine Tokyo Medical University Hachioji Medical Center Tokyo Japan; ^2^ Emergency and Disaster Medicine Tokyo Medical University Tokyo Japan

**Keywords:** Catheter‐based technique, coagulopathy, interventional radiology, intra‐aortic balloon occlusion, resuscitative endovascular balloon occlusion of the aorta, shock

## Abstract

Trauma patients with uncontrolled hemorrhage encountering coagulopathy are often associated with poor outcome. Recently, the concept of damage control interventional radiology, which focuses on “speedy stoppage of bleeding” by interventional radiology among trauma patients with hemodynamic instability and acute traumatic coagulopathy, was proposed as an alternative to damage control surgery. N‐butyl cyanoacrylate (NBCA) has been used as a liquid embolic agent in various non‐traumatic situations, where it has been shown to have a high technical success rate and low recurrent bleeding rate, especially in patients with coagulopathy. In this case, we treated a young patient with hemodynamic instability caused by a high‐grade hepatic injury, who underwent arterial embolization (AE) using NBCA assisted with resuscitative endovascular balloon occlusion of the aorta and achieved successful hemostasis. A review of published works using PUBMED was carried out, and 10 published reports involving 23 trauma patients who underwent AE using NBCA were identified. Among them, only four reports involving five trauma patients with torso visceral injuries were identified. Three of five patients who were hemodynamically unstable underwent AE using NBCA, resulting in the stabilization of hemodynamics. We concluded that AE with resuscitative endovascular balloon occlusion of the aorta as a damage control interventional radiology procedure might be acceptable for the hemodynamically unstable hepatic injury, and NBCA could be one of the effective hemostatic agents for this purpose, in cases of trauma‐induced coagulopathy.

## Background

Hemorrhage is a main cause of death in patients with hemorrhagic shock admitted to the emergency department, and trauma is the most common cause of massive hemorrhage in the acute care setting. The development of endovascular equipment and embolic agents has contributed to the acceptance of endovascular treatment for hemorrhage as an effective and safe method. Recently, n‐butyl cyanoacrylate (NBCA) has been used as a liquid embolic agent in various non‐traumatic situations, where it has been shown to have a high technical success rate and low recurrent bleeding rate, especially in patients with coagulopathy.^1^, ^2^ There are also a few reports of successful use of NBCA in trauma patients, thus it appears to be an effective method in cases of severe trauma.^3^ We treated a young patient with hemodynamic instability caused by a high‐grade hepatic injury, who underwent arterial embolization (AE) using NBCA assisted with resuscitative endovascular balloon occlusion of the aorta (REBOA). In addition, we reviewed published works to provide a summary of the experience data.

## Consent

The ethics committee of Tokyo Medical University Hachioji Medical Center approved the design of this study. Written informed consent was obtained from the patient for publication of this case report and accompanying images.

## Review

### Case

A 19‐year‐old woman was admitted to our emergency center after a motor vehicle accident at night. Her previous medical history was unremarkable. Physical examination revealed the following: Glasgow Coma Scale score, E2V3M5; blood pressure (BP), 89/56 mmHg; heart rate, 126 beats/min; and respiratory rate, 25 breaths/min. She had a degloving injury of her left lower leg. She was hemodynamically unstable and immediately received initial trauma resuscitation on the basis of Advanced Trauma Life Support guidelines.^4^ Abdominal sonography revealed massive fluid collection in the abdomen, and chest X‐ray revealed bilateral pneumothorax with lung contusion. The patient's platelet counts was 11.2 × 10^4^/μL (reference range, 15–35 × 10^4^/μL), hemoglobin was 9.1 g/dL (12.0–16.0 g/dL), hematocrit was 21% (35–48%), base excess was −11.7 mmol/L (−2.2 to 1.2 mmol/L), prothrombin time (PT) was 45% (80–100%), fibrin degradation product was 174.2 μg/mL (<5.0 μg/mL), and D‐dimer was 42.46 μg/mL (<0.5 μg/mL). The international normalized ratio of PT was 1.81.

**Figure 1:**
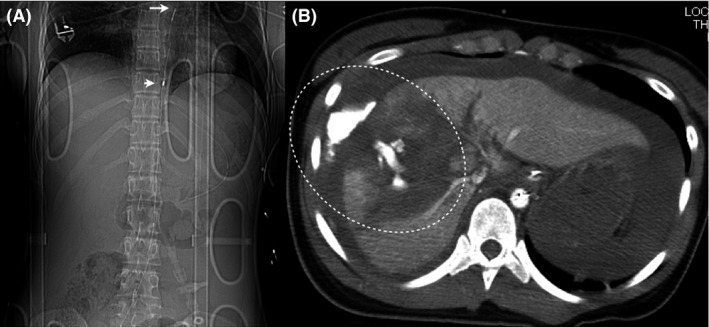
Computed tomography scout image of a 19‐year‐old woman with hemodynamic instability caused by a high‐grade hepatic injury revealed the position of the intra‐aortic balloon occlusion catheter. A, The tip of the catheter (long arrow) and proximal end of the balloon (short arrow) are shown. B, Contrast‐enhanced computed tomography (CT) revealed the extravasation of contrast medium from hepatic trauma (circle). Classified as CT grade IV according to the American Association for the Surgery of Trauma CT scale.

Although Ringer's lactate and blood transfusion were rapidly administered, she did not respond to fluid resuscitation, and her BP deteriorated. Thus, we undertook REBOA prior to hemostasis. After the blind insertion of a 7‐Fr sheath into the right femoral artery, a balloon occlusion catheter was inserted into the aorta in Zone I according to the recommendations of Stannard *et al*.^5^ under ultrasonographic guidance (Fig. 1A). Subsequently, her systolic BP increased to 125 mmHg after REBOA.

**Figure 2:**
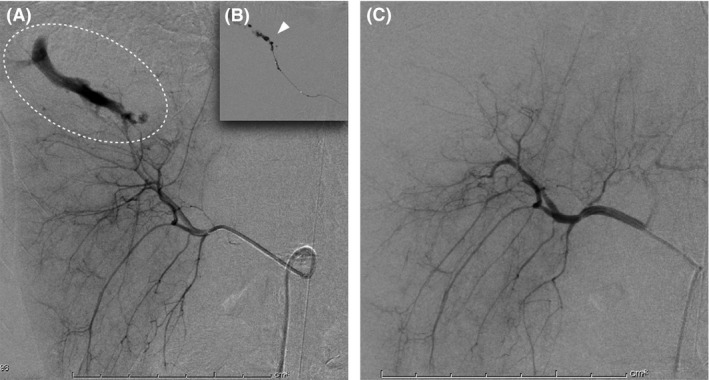
A, Angiography revealed bleeding from a peripheral branch arising from the right anterior hepatic artery (circle) in a 19‐year‐old woman with hemodynamic instability caused by a high‐grade hepatic injury. B, Injection of n‐butyl cyanoacrylate (arrow head). C, Angiography confirmed that endovascular hemostasis had been achieved.

**Figure 3:**
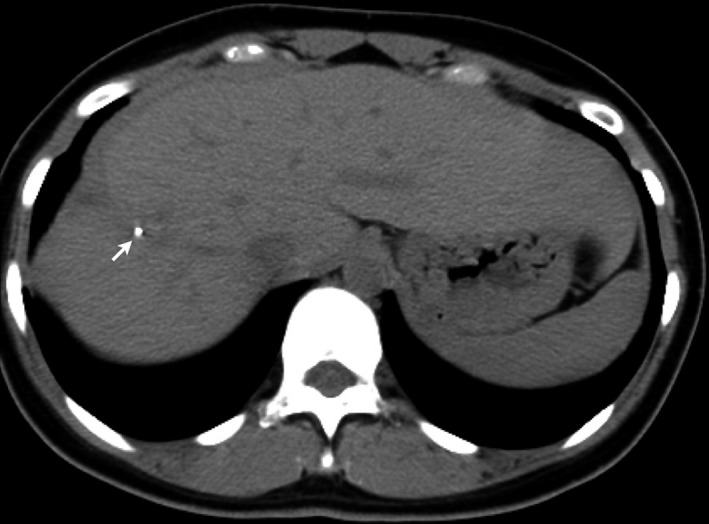
Follow‐up computed tomography in a 19‐year‐old woman 1 year after a high‐grade hepatic injury caused hemodynamic instability. Arrow indicates injection site of n‐butyl cyanoacrylate.

Computed tomography (CT) revealed a laceration of the liver with marked extravasation of the contrast medium, classified as CT grade IV according to the American Association for the Surgery of Trauma CT scale,^6^ and fluid collection (Fig. 1B). Injury Severity Score was 41. In the angiography suite, a 4‐Fr guiding sheath catheter (Parent; Medikit, Tokyo, Japan) was placed into the celiac artery through the left femoral artery, and angiography revealed marked bleeding from a peripheral branch arising from the right anterior hepatic artery (AHA) (Fig. 2A). Superselective catheterization using a microcatheter (SL‐10; Stryker, Tokyo, Japan) in a triaxial system was undertaken into the AHA, followed by the careful injection of 0.5 mL NBCA (Histoacryl; Braun, Melsungen, Germany), which was mixed with iodized oil (Lipiodol; Andre Guerbet, Aulnay‐sous‐Bois, France) at a ratio of 1:2 (Fig. 2B). Angiography confirmed that endovascular hemostasis had been achieved (Fig. 2C). The resuscitation interval between hospital arrival and insertion of REBOA was 50 min, and the procedure interval for hemostasis after the insertion of the guiding sheath catheter was 25 min. The total REBOA balloon handling time was 62 min. A tube drainage was carried out for intra‐abdominal hematoma after AE. A total of 2,800 mL red blood cells and 1,920 mL fresh frozen plasma were given within 24 h. The patient underwent skin graft of her lower leg because of a degloving injury, and was discharged after 3 months. The patient continues to remain symptom‐free 1 year after injury (Fig. 3).

### Review of published works

A search using PUBMED to include “n‐butyl cyanoacrylate” and “trauma” or “injury” was carried out. Original articles and case reports published in English were reviewed, and follow‐up references listed were further investigated. Ten published reports involving 23 trauma patients who underwent AE using NBCA were identified.^7^, ^8^, ^9^, ^10^, ^11^, ^12^, ^13^, ^14^, ^15^, ^16^ Among them, eight patients had a history of blunt trauma, and the remaining patients had a history of penetrating trauma. Three of the blunt trauma patients (hemothorax, 1; splenic injury, 1; and colon injury, 1) and two of the penetrating trauma patients (renal injury, 2) had torso visceral injuries, as shown in Table 1.^7^, ^8^, ^10^, ^14^ Table 1 shows the clinical characteristics of the patients involved. The three patients with blunt torso visceral injuries showed hemodynamic instability and underwent AE using NBCA, resulting in immediate hemodynamic stabilization. Other penetrating injuries were successfully treated with AE. Complications, including recanalization and intestinal ischemia, were reported in torso visceral injuries, and reflux (*n* = 2) and thrombosis (*n* = 1) were reported in penetrating lower leg injuries.

**Table 1:** Review of published works regarding trauma patients treated by n‐butyl cyanoacrylate (NBCA)

Authors	Year	*n*	Age	Sex	Clinical presentation	Type of trauma	Hemodynamic instability	Targeted artery	Angiography findings	Results	NBCA : lipiodol	Volume of NBCA, mL
Aoki *et al*.^7^	2015	1	81	F	Hemothorax/pelvic fracture	Blunt	Yes	Inferior phrenic artery	CM extravasation	Success	1: 3	NA
Ishikawa *et al*.^8^	2011	1	70	F	Hemoperitoneum	Blunt	Yes	Splenic artery	Pseudoaneurysm/CM extravasation	Recanalization	1: 2	0.3
Rados *et al*.^9^	2010	1	20	M	Perineal trauma/priapism	Blunt	No	Cavernous artery	Pseudoaneurysm/AVF	Success	NA	NA
Toyoda *et al*.^10^	2009	1	30	M	Retroperitoneal hematoma	Blunt	Yes	IMA (sigmoid artery)	CM extravasation	Intestinal ischemia	1: 1.5	NA
Lopera *et al*.^11^	2008	1	32	F	Refractory toe ulcer/leg edema	Penetrating	No	Plantar arch	AVF	Success	NA	NA
Mavili *et al*.^12^	2007	12	36	M	Lower limb trauma	Penetrating	No	Deep femoral artery	Pseudoaneurysm	Success	1:2	0.2–0.6
			16	M	Lower limb trauma	Penetrating	No	Deep femoral artery	Pseudoaneurysm/AVF	Success	1:2	0.2–0.6
			20	M	Lower limb trauma	Penetrating	No	Inferior gluteal artery	Pseudoaneurysm	Reflux	1:2	0.2–0.6
			33	M	Lower limb trauma	Penetrating	No	Peroneal artery	Pseudoaneurysm	Success	1:2	0.2–0.6
			33	M	Lower limb trauma	Penetrating	No	Superficial femoral artery	Pseudoaneurysm	Thrombosis	1:2	0.2–0.6
			44	M	Lower limb trauma	Penetrating	No	Deep femoral artery	Pseudoaneurysm	Inguinal hematoma	1:2	0.2–0.6
			25	M	Lower limb trauma/recurrent bleeding	Penetrating	No	Deep femoral artery	Pseudoaneurysm/AVF	Success	1:2	0.2–0.6
			19	M	Lower limb trauma/persistent pain/mass	Penetrating	No	Deep femoral artery	Pseudoaneurysm/AVF	Success	1:2	0.2–0.6
			14	M	Lower limb trauma	Penetrating	No	Deep femoral artery	Pseudoaneurysm	Reflux	1:2	0.2–0.6
			45	M	Lower limb trauma	Penetrating	No	Peroneal artery	Pseudoaneurysm	Success	1:2	0.2–0.6
			17	F	Lower limb trauma	Penetrating	No	Anterior tibial artery	Pseudoaneurysm	Success	1:2	0.2–0.6
			42	M	Lower limb trauma	Penetrating	No	Peroneal artery	AVF	Success	1:2	0.2–0.6
Kim *et al*.^13^	2007	1	53	M	Perineal trauma/priapism	Blunt	No	Cavernous artery	AVF	Success	NA	NA
Cantasdemir *et al*.^14^	2003	2	33	M	Hematuria	Penetrating	No	Renal artery (pole interlobar artery)	Pseudoaneurysm	Success	1:2	0.7
			33	M	Hematuria	Penetrating	No	Renal artery (pole interlobar artery)	Pseudoaneurysm	Success	1:2	0.9
Numan *et al*.^15^	1996	1	72	M	Pelvic fracture/priapism	Blunt	No	Cavernous artery	AVF	Success	1:3	0.5
Alvarez *et al*.^16^	1994	2	21	M	Perineal trauma/priapism	Blunt	No	Cavernous artery	AVF	Success	NA	0.6
			33	M	Perineal trauma/priapism	Blunt	No	Cavernous artery	AVF	Success	NA	0.8
Present case	2016	1	19	F	Hemoperitoneum	Blunt	Yes	Hepatic artery	CM extravasation	Success	1:2	0.5

AVF, arteriovenous fistula; CM, contrast medium; F, female; IMA, inferior mesenteric artery; M, male; *n*, number of patients; NA, not available.

## Discussion

The primary aim of resuscitation is to stop the source of hemorrhage and restore normal hemodynamics because massive hemorrhage can rapidly progress to death. Patients with uncontrolled hemorrhage encountering coagulopathy are often associated with poor outcome. From our review of existing reports, it was evident that there are only a very limited number of publications related to AE using NBCA for trauma, and only five cases with torso visceral injuries (blunt trauma, 3; and penetrating trauma, 2) were retrieved. Three of five patients with blunt trauma who were hemodynamically unstable underwent AE using NBCA, resulting in the stabilization of hemodynamics. To the best of our knowledge, the present study represents the first report describing the utility of AE using NBCA for a patient who had hemodynamic instability with a high‐grade hepatic injury.

Recently, the concept of damage control interventional radiology (DCIR), which focuses on “speedy stoppage of bleeding” by any possible means among trauma patients with hemodynamic instability and acute traumatic coagulopathy, was proposed as an alternative to damage control surgery in Japan.^3^ The main concern associated with DCIR is to maintain or re‐establish normal hemodynamics to contain the ongoing hemorrhage using minimally invasive interventional radiology (IR) as soon as possible. Our belief that acute vascular IR should be carried out by IR‐trained acute care specialists in acute trauma resuscitation with or without hemodynamic instability, and the details of treatment indications and protocols have been previously described.^17^ The concept of DCIR supports our belief, and we conduct training in the radiology department to practice handling these embolic agents, which may help to achieve successful hemostasis in future trauma resuscitation.

There has been marked progress in the development of embolic agents over the past decade and there are a variety of materials now available, including metallic coils, gelatin sponge particles, and liquid and nitinol plugs. Among them, gelatin sponge particles are classified as temporary embolic agents, and coils, NBCA, and nitinol plugs are classified as permanent embolic agents.^18^ Gelatin sponge particles or coils involve physical blocking of blood flow with thrombus formation around the materials in the vascular lumen. N‐butyl cyanoacrylate is routinely used by mixing with iodized oil to make it radiopaque; when it comes into contact with cations, it polymerizes to become a solid substance in the vascular lumen. Furthermore, the operator can adjust the extent of AE by changing the mixing ratio.

The effectiveness of embolization using NBCA, gelatin sponges, or microcoils remains controversial. The use of gelatin sponges is popular because of their ease of handling in both traumatic and non‐traumatic clinical settings. N‐butyl cyanoacrylate is increasingly being used because of its highly penetrable liquid nature in various non‐traumatic situations.^1^, ^19^, ^20^, ^21^, ^22^, ^23^, ^24^ Won *et al*.^19^ successfully treated visceral pseudoaneurysms located in the gastroduodenal, pancreaticoduodenal, dorsal pancreatic, jejunal, colic, splenic, renal, and hepatic arteries with AE using NBCA. Kim *et al*.^20^ also reported the efficacy of AE using NBCA for renal bleeding with a high success rate (100%). Arterial embolization using NBCA is effective in the management of acute non‐variceal gastrointestinal hemorrhage with or without hemodynamic instability, and the incidence rate of intestinal ischemia was reportedly low.^1^, ^22^, ^23^, ^24^ Although AE using microcoils requires considerable time to carry out, NBCA is a much more rapid intervention for hemostasis than other materials in a coagulopathic condition with PT international normalized ratio >1.5.^18^ A recent study reported that NBCA was more effective in an animal model of severe acute traumatic coagulopathy with activated clotting time >400 s.^25^ Thus, AE using NBCA appears to be safe and effective among patients with acute bleeding from various visceral arteries or peripheral arteries.

Resuscitative endovascular balloon occlusion of the aorta has been found to successfully elevate central blood pressure in patients with hemorrhagic shock in various clinical settings.^26^, ^27^, ^28^, ^29^ Therefore, we used REBOA procedures in patients who had hemodynamic instability and an inability to remain normotensive following resuscitation. This procedure was not routinely used and the details of REBOA indications and procedures have been previously described.^26^ Ogura *et al*. reported their clinical experiences of hemodynamically unstable patients with abdominal solid organ injuries managed non‐operatively using AE with REBOA as an adjunct. Although more evidence is needed, AE with REBOA as a DCIR procedure might be acceptable for the hemodynamically unstable hepatic injury, and NBCA could be one of the effective hemostatic agents for this purpose, in cases of trauma‐induced coagulopathy.

Some training is required in the use of NBCA because of the risk of ischemic complications or reflux, and the need to handle it safely. In the review of published works, recanalization and intestinal ischemia were reported among patients with torso visceral injuries. These were common disadvantages of AE using NBCA. To prevent recanalization, Ishikawa *et al*.^8^ suggested that interventional radiologists should determine the appropriate concentration and volume of the NBCA/lipiodol mixture based on blood flow, and the range of the parent artery to be occluded. Concerning the procedures themselves, injection of NBCA had to be achieved rapidly in order to avoid adherence of the catheter to the glue, and the catheter should be retrieved with aspiration after the injection.

## Conclusion

Arterial embolization using NBCA appears to be safe and effective among patients with both hemodynamic instability and acute traumatic coagulopathy caused by severe visceral trauma.

## Conflict of Interest

None Declared.
